# Barriers to continuity of outpatient infusion-port maintenance among patients with cancer: a single-center cross-sectional study in China

**DOI:** 10.3389/fpubh.2026.1877650

**Published:** 2026-06-26

**Authors:** Yongchao Zhou, Yuxiu Xi, Xiao Zhu, Weijing Ni

**Affiliations:** Intravenous Therapy and Nursing Clinic, Affiliated Hospital of Jiangnan University, Wuxi, China

**Keywords:** cancer supportive care, financial toxicity, infusion port, maintenance continuity, outpatient oncology, totally implantable venous access port

## Abstract

**Background:**

Totally implantable venous access ports require timely outpatient maintenance to preserve long-term device safety; however, missed or delayed visits may reflect financial, health system, behavioral, psychosocial, and knowledge-related barriers. This study assessed the prevalence, determinants, and patient-reported consequences of discontinuous outpatient infusion-port maintenance among cancer patients in China.

**Methods:**

This hospital-based observational analytical questionnaire study included adult patients with cancer who had an indwelling infusion port or comparable long-term venous access device. Discontinuous maintenance was defined as at least one missed or delayed scheduled maintenance visit during the preceding 6 months. Five prespecified barrier domain scores were evaluated: knowledge deficit, health system barriers, financial barriers, behavioral barriers, and psychosocial barriers. Modified Poisson regression with robust standard errors was used to estimate the prevalence ratios for discontinuous maintenance, and ordinal logistic regression was used to assess the severity of missed visits, satisfaction, and future maintenance intention.

**Results:**

A total of 2,710 patients were included, of whom 1,076 (39.7%) reported discontinuous maintenance. Financial burden was the most common reason for the most recent missed or delayed visit (32.9%), followed by transportation difficulties (20.1%), fear or anxiety (18.9%), and lack of knowledge about maintenance timing (12.1%). In fully adjusted models, discontinuity was independently associated with financial barriers (adjusted prevalence ratio [aPR], 1.59; 95% CI, 1.47–1.72), knowledge deficit (aPR, 1.57; 95% CI, 1.44–1.72), health-system barriers (aPR, 1.40; 95% CI, 1.28–1.53), psychosocial barriers (aPR, 1.33; 95% CI, 1.22–1.44), and behavioral barriers (aPR, 1.30; 95% CI, 1.20–1.41). Discontinuity increased across the total barrier score quartiles from 13.0 to 68.9% (*p* for trend < 0.001), indicating a strong cumulative barrier burden gradient. Adding barrier domains improved model discrimination from an AUC of 0.646 to 0.776. Discontinuous maintenance was also associated with more unscheduled port-related visits (22.7% vs. 5.5%; *p* < 0.001), lower satisfaction, and weaker future attendance intention.

**Conclusion:**

Discontinuous infusion-port maintenance was common and independently associated with multiple modifiable barriers, supporting integrated interventions that combine patient education, appointment coordination, financial navigation, transport support, and reminder systems.

## 1 Introduction

Cancer care increasingly depends on durable vascular access systems that can support repeated chemotherapy, targeted therapy, immunotherapy, parenteral nutrition, blood sampling, and supportive infusions without the cumulative harm associated with repeated peripheral venipunctures. In 2022, approximately 20 million new cancer cases and 9.7 million cancer deaths occurred worldwide, with China alone accounting for an estimated 4.82 million incident cancer cases and 2.57 million cancer deaths, underscoring the scale of long-term oncology service demand in the country (1, 2). Totally implantable venous access ports (TIVAPs), also described as implantable port catheters, have become a central component of contemporary cancer treatment because they provide stable central venous access, improve patient comfort, and may reduce several catheter-related complications compared with externally exposed vascular access devices when appropriately inserted, accessed, and maintained (3–6).

The clinical value of an infusion port depends not only on technically successful implantation but also on the continuity of maintenance after hospital discharge and between treatment cycles. International standards and Chinese expert guidance emphasize standardized flushing, locking, aseptic access, complication surveillance, patient education, and quality-control pathways as core elements of safe TIVAP management (7–9). Recent evidence has also challenged rigid monthly flushing schedules in selected off-treatment populations, with observational studies and meta-analyses suggesting that longer maintenance intervals may be feasible in carefully monitored patients; however, such findings should not be interpreted as support for unplanned discontinuity, because delayed or missed care may occur in patients with poorer knowledge, limited service access, or higher complication risk (10–13). Thus, the public health question is not merely how often a port should be flushed under controlled conditions, but whether patients can reliably access timely, competent, and coordinated outpatient maintenance in routine practice.

Outpatient port maintenance continuity is particularly relevant in China, where expanding cancer survivorship and prolonged systemic therapy intersect with regional inequities in specialist nursing capacity, insurance coverage, and referral infrastructure (2, 12, 14). Community health facilities that absorb maintenance demand during periods of tertiary outpatient crowding or service disruption vary widely in trained personnel, equipment, and institutional capacity to manage implanted ports (15, 16). For individual patients, the maintenance pathway is further shaped by financial cost, transport feasibility, appointment availability, health literacy, psychosocial readiness, and caregiver support—factors that are modifiable yet unevenly distributed across sociodemographic strata (17, 18).

Despite this public health significance, patient-level continuity of outpatient port maintenance has received substantially less empirical attention than device insertion, flushing protocols, or catheter-related complications. Chinese studies have advanced TIVAP self-management measurement and guideline development; however, structured evidence on why patients miss, delay, or discontinue outpatient maintenance visits—and which barriers independently predict discontinuity—remains limited (4, 6, 15, 16, 19). This gap restricts the capacity of oncology services to design targeted interventions such as reminder systems, nurse-led navigation, decentralized maintenance clinics, and risk-stratified follow-up pathways (20, 21). Therefore, this single-center questionnaire study was designed to identify barriers to the continuity of outpatient infusion-port maintenance services among cancer patients in China. By assessing sociodemographic, clinical, knowledge-related, structural, financial, behavioral, psychosocial, and care continuity domains, this study aimed to quantify the prevalence of disrupted maintenance and determine which modifiable barriers are independently associated with missed or delayed follow-up. These findings may provide evidence for patient-centered port maintenance pathways that are clinically safe, operationally feasible, and responsive to real-world constraints faced by patients receiving long-term cancer care.

## 2 Methodology

### 2.1 Study design and setting

This hospital-based observational analytical questionnaire study was designed to evaluate the barriers to the continuity of outpatient totally implantable venous access port maintenance among patients with cancer. The investigation was conducted at the Affiliated Hospital of Jiangnan University, Wuxi, China, between February 2023 and October 2025. With no changes to clinical protocols, staffing, scheduling, or reimbursement during this period, the cohort reflects a stable institutional practice environment. The study conceptualized port maintenance as a longitudinal component of outpatient supportive oncology care rather than as an isolated technical procedure, because long-term device safety depends on timely flushing, clinical inspection, complication surveillance, patient education, and access to trained personnel after discharge and between treatment cycles (4, 22, 23).

The conceptual framework integrated patient-level, health system, financial, behavioral, and psychosocial determinants of care continuity. This framework was selected because missed or delayed maintenance may reflect not only individual adherence but also structural constraints, including appointment availability, transportation burden, insurance coverage, clinic waiting time, service coordination, and the availability of community-based maintenance support. The primary analytical objective was to identify modifiable barriers associated with discontinuous port maintenance, whereas the secondary objectives evaluated delay severity, missed-visit burden, port-related unscheduled care, satisfaction with maintenance services, and intention to attend future maintenance visits on time (23, 24).

### 2.2 Study population and eligibility criteria

The source population comprised adult patients with a confirmed cancer diagnosis who had an indwelling venous access port or related long-term central venous access device and were expected to undergo periodic outpatient maintenance according to institutional practices. Eligible participants were aged ≥18 years, had an implanted port or a comparable long-term venous access device at the time of the survey, had sufficient clinical stability to complete the questionnaire, and were able to provide informed consent and reliable self-reported information regarding recent maintenance behavior.

Patients were excluded if they were unable to complete the questionnaire because of severe acute illness, cognitive impairment, communication barriers, or other conditions that precluded reliable responses. Patients were also excluded from the analytical dataset if the core outcome data required to classify maintenance continuity were missing, inconsistent, or not interpretable after data cleaning. For patients whose ports had been implanted for <6 months, the maintenance history was interpreted according to the available period since implantation; however, the primary continuity variable required sufficient information to determine whether a scheduled maintenance visit had been missed or delayed.

The adequacy of the analytical sample was evaluated using a single-proportion sample size framework to estimate the prevalence of discontinuous maintenance. Assuming a conservative expected prevalence of 50%, a 95% confidence level, and a 2.0% absolute margin of error, the minimum required sample size was calculated using the formula *n* = *Z*^2^*p*(1 − *p*)/*d*^2^, yielding 2,401 participants before accounting for incomplete responses. The final analytical sample exceeded this minimum requirement, providing sufficient precision for prevalence estimation and adequate statistical stability for the multivariate modeling of prespecified barrier domains and covariates.

### 2.3 Questionnaire development and data collection

Data were collected using a structured, 50-item questionnaire developed to capture clinically relevant and public health-oriented determinants of outpatient port maintenance continuity. The questionnaire included sociodemographic, clinical, and port-related characteristics; knowledge and awareness of port maintenance; structural and healthcare system barriers; financial barriers; patient-related and behavioral barriers; psychosocial barriers; and continuity-of-care outcomes. The instrument was designed to be concise enough for routine outpatient administration while retaining sufficient domain coverage for reliability testing, multivariable modeling, and service-improvement interpretation.

The questionnaire was developed by reviewing relevant oncology vascular-access practice standards, Chinese port-maintenance guidelines, and empirical literature on outpatient central venous access maintenance, patient self-management, and continuity of supportive cancer care. Content validity was assessed by a multidisciplinary expert panel comprising oncology nurses, medical oncologists, public health researchers, and clinical methodology specialists, who reviewed item relevance, wording clarity, domain coverage, and feasibility for outpatient administration (25). The instrument was pilot-tested in 20 patients to evaluate comprehension, completion time, and acceptability; minor wording refinements were made before formal data collection without altering the prespecified domain structure.

Sociodemographic variables included age, sex, place of residence, marital status, educational attainment, employment status, monthly household income, and type of medical insurance. Clinical and device-related variables included primary cancer diagnosis, current treatment status, cancer stage, port type, duration since port implantation, and history of previous port-related complications. Barrier-related questionnaire items used ordered response categories, primarily 5-point Likert-type scales. Items were worded to capture practical barriers encountered by patients during routine maintenance, including difficulty obtaining appointments, long waiting times, distance to the maintenance clinic, transportation barriers, cost burden, loss of work or caregiver time, forgetting scheduled visits, reduced perceived need after treatment interruption or completion, anxiety about complications, fear of pain, inadequate family support, and prior negative healthcare experiences.

Trained personnel administered paper questionnaires in outpatient oncology or port-maintenance settings using standardized instructions. To mitigate literacy barriers, staff read questions aloud and provided clarification as needed, though patients requiring extensive help may still be underrepresented. Readability wasn’t quantified using a standard index; instead, item clarity was refined based on feedback from a 20-patient pilot test (median completion time wasn’t recorded). Participants evaluated their experience over the preceding 6 months, or since implantation if the port was newer. Because the primary outcome relied on a retrospective self-report, a 6-month recall window was selected to balance the need to capture meaningful maintenance behavior against the risk of recall error. Responses were checked for completeness at the time of collection where feasible, without influencing participant answers. All questionnaire data were subsequently coded into a structured dataset using prespecified variable definitions.

### 2.4 Outcome definitions

The primary outcome was discontinuous outpatient port maintenance, defined as at least one missed or delayed scheduled maintenance visit beyond the recommended maintenance date during the preceding 6 months (11). Participants reporting no missed or delayed maintenance visits were classified as having continuous maintenance, whereas participants reporting one or more missed or delayed visits were classified as having discontinuous maintenance. This binary outcome was coded as 0 for continuous maintenance and 1 for discontinuous maintenance.

Delay severity was assessed using the longest self-reported delay beyond the recommended maintenance date. The categories included no delay, <1 week, 1–2 weeks, >2 weeks, and not sure. Missed-visit severity was assessed as an ordinal variable representing the increasing frequency of missed or delayed maintenance visits during the preceding 6 months and categorized as 0, 1, 2, or ≥3 missed or delayed visits.

Port-related safety outcomes included unscheduled hospital visits for port-related symptoms or concerns, and emergency care or hospitalization attributable to port-related problems. These outcomes were considered secondary indicators of clinically relevant disruption or complication-related service use.

Patient-reported service outcomes included satisfaction with outpatient port maintenance services and future intention to attend maintenance visits on time. Satisfaction was measured using a five-level ordered scale ranging from very satisfied to very dissatisfied for ordinal modeling, with categories coded so that higher values represented worse satisfaction. Future intention was measured using a four-category ordered scale: will attend, probably will attend, unsure, and probably will not attend. Categories were coded so that higher values represented progressively weaker intentions to attend maintenance visits on time.

### 2.5 Exposure variables and covariates

The principal exposure variables were five prespecified barrier-domain scores: knowledge deficit, health system barriers, financial barriers, behavioral barriers, and psychosocial barriers. The knowledge-deficit domain was derived from items assessing awareness of recommended maintenance intervals, understanding of flushing requirements when treatment was paused, awareness of risks associated with delayed maintenance, recognition of warning signs, knowledge of whom to contact during port-related symptoms, and receipt of clear maintenance education. Positively worded knowledge items were reverse-coded so that higher scores represented greater knowledge deficit.

The health system barrier domain included items addressing appointment convenience, appointment-slot availability, outpatient service hours, waiting time, distance from home to the clinic, transportation difficulty, difficulty identifying the appropriate department, and perceived coordination between oncology departments, outpatient clinics, nurses, and community services. Positively worded system performance items were reverse-coded so that higher scores consistently indicated greater health system barriers.

The financial barrier domain captured direct medical costs, indirect nonmedical costs, loss of work time or caregiver work time, adequacy of insurance coverage, and overall financial pressure causing delays or avoidance of maintenance. Items indicating affordability or adequate insurance coverage were reverse-coded where appropriate. The behavioral barrier domain included forgetfulness, delays during weakness or illness, reduced perceived importance when treatment was paused or completed, time-arrangement difficulty, and maintenance-related behavioral constraints. The psychosocial barrier domain included anxiety about complications, fear of pain or discomfort, previous negative healthcare experiences, lack of family or caregiver support, and privacy, body image, or embarrassment concerns.

Each item was scored on an ordinal scale and recoded so that higher scores uniformly represented greater barrier burden. Domain scores were calculated as the means of valid items within each domain, provided that sufficient non-missing items were available for that domain according to the prespecified scoring rules. A total barrier score was calculated as the mean of the five domain scores and was used for dose–response and sensitivity analyses, whereas individual domain scores were retained for etiologically interpretable multivariate models.

Covariates were selected *a priori* based on their clinical plausibility and relevance to outpatient service access. Age was analyzed as a continuous variable and scaled per 10-year increase in the regression models. Residences were categorized as urban, suburban, or rural. Educational attainment and monthly household income were modeled as ordered, categorical variables. Additional covariates included sex, insurance type, primary cancer diagnosis, current treatment status, cancer stage, port type, duration since port implantation, and history of port-related complications. Insurance type was categorized as Urban Employee Basic Medical Insurance (UEBMI; a mandatory, payroll-linked contributory scheme covering employed urban workers and retirees), Urban–Rural Resident Basic Medical Insurance (URRBMI; a voluntary, government-subsidized scheme consolidating the former urban resident insurance and new rural cooperative medical programs, covering non-employed urban residents, rural residents, and students), commercial or supplementary private insurance, or self-pay and other coverage.

### 2.6 Scale evaluation and data quality assessment

Before the domain scores were used as analytical predictors, the measurement properties of each barrier domain were evaluated. Internal consistency was assessed using Cronbach’s alpha and McDonald’s omega, because alpha provides a conventional estimate of scale reliability, whereas omega is less dependent on strict tau-equivalence assumptions and is often more appropriate for multidimensional applied questionnaires (26). Corrected item-total correlations were examined to assess whether individual items contributed coherently to their prespecified domains. Domain score distributions were summarized using means, standard deviations, medians, interquartile ranges, and observed ranges.

A Cronbach’s alpha value of 0.70 or higher was conventionally considered acceptable for mature scales; however, lower alpha values were not interpreted as automatic evidence of unusable measurement because several domains intentionally captured heterogeneous barriers rather than narrowly redundant symptoms. Therefore, McDonald’s omega, corrected item-total correlations, and conceptual coherence were considered jointly when deciding whether to retain prespecified domains. This approach was particularly important for behavioral and psychosocial domains, where items were expected to represent related but non-identical mechanisms of interrupted maintenance.

Interdomain associations were examined using Spearman’s correlation coefficients because barrier-domain scores were derived from ordinal questionnaire responses and were not assumed to follow a normal distribution. A correlation coefficient >0.70 was prespecified as evidence of potentially problematic overlap or multicollinearity between domains. Variance inflation factors were assessed in multivariable models to further evaluate collinearity among covariates and barrier scores.

Data quality procedures included assessment of missingness, impossible values, inconsistent response patterns, and outcome derivation logic. Age- and duration-related variables were checked for plausible ranges. Outcome variables were checked to ensure that the missed-visit frequency, delay severity, and reason for missed or delayed maintenance were logically consistent. Records with missing or uninterpretable primary outcome information were excluded from the primary analyses, whereas item-level missingness within barrier domains was handled according to prespecified scoring rules.

### 2.7 Statistical analysis

All analyses were conducted using Python 3.11 with pandas 2.2, numpy 1.26, scipy 1.11, statsmodels 0.14, scikit-learn 1.4, and matplotlib 3.8. Analyses were performed using the cleaned analytical dataset after application of eligibility criteria, coding rules, and derived-variable definitions. Descriptive statistics were used to characterize the study population overall and by port maintenance continuity status. Continuous variables were summarized as mean and standard deviation or median and interquartile range, depending on distributional characteristics. Categorical variables were summarized as frequencies and percentages. Between-group comparisons used Welch t tests or nonparametric rank-based tests for continuous or ordinal variables where appropriate, and Pearson chi-square tests or Fisher exact tests for categorical variables when expected cell counts were small. These comparisons were interpreted descriptively and were not used as the sole basis for covariate selection. The primary regression outcome was discontinuous port maintenance. Because the outcome was expected to be common, prevalence ratios were preferred over odds ratios to improve interpretability (27). Univariable associations were first estimated using modified Poisson regression with robust standard errors. Multivariable modified Poisson regression was then used to estimate adjusted prevalence ratios and 95% confidence intervals. The principal adjusted model included age, sex, residence, education, income, insurance type, clinical covariates, device-related covariates, prior port-related complication history, and the five barrier-domain scores. Effect estimates were reported as prevalence ratios or adjusted prevalence ratios with 95% confidence intervals and 2-sided *p* values.

Subgroup analyses evaluated whether the association between financial barriers and discontinuous maintenance differed by residence, income, and education. Stratum-specific adjusted prevalence ratios were estimated, and multiplicative interaction terms were used to test effect modification. Interaction analyses were interpreted cautiously because subgroup models may be less precise and were intended to identify potentially vulnerable populations rather than establish causal heterogeneity. Dose–response analysis examined the proportion of discontinuous maintenance across quartiles of the total barrier score. A test for trend was performed by modeling barrier-score quartile as an ordered variable. This analysis was used to evaluate whether increasing cumulative barrier burden corresponded to progressively greater disruption in port maintenance continuity.

Missed-visit severity was analyzed using ordinal logistic regression, with the ordinal outcome representing increasing frequency of missed or delayed maintenance visits. Predictors included age, sociodemographic covariates, clinical and port-related covariates, and the five barrier-domain scores. The proportional odds assumption was assessed where feasible, and results were reported as proportional odds ratios with 95% confidence intervals. Satisfaction with port maintenance services and future intention to attend maintenance on time were evaluated using parallel ordinal logistic regression models. For each outcome, the first model included covariates and the five barrier-domain scores. A second model additionally included discontinuous maintenance to evaluate whether maintenance discontinuity attenuated the association between barrier burden and patient-reported outcomes. Attenuation was calculated on the log proportional-odds-ratio scale using the percentage reduction in the log proportional odds ratio after discontinuous maintenance was added to the model. This attenuation analysis was interpreted as exploratory and not as formal causal mediation, because the cross-sectional design does not establish temporal ordering among barriers, discontinuity, satisfaction, and future intention.

Model discrimination for discontinuous maintenance was assessed using receiver operating characteristic curves. A model including demographic, clinical, and barrier-domain variables was compared descriptively with a demographic-only model by estimating the area under the receiver operating characteristic curve. Predictive performance was presented as supportive evidence of incremental model information and was not used to infer causality.

A sensitivity analysis restricted the primary modified Poisson model to participants whose port duration was at least 6 months. This analysis was conducted because patients with newly implanted ports had a shorter opportunity window in which to miss or delay scheduled maintenance visits. Consistency between the main model and this restricted analysis was interpreted as evidence that associations were not driven solely by differential opportunity for discontinuity among patients with more recent port placement. All statistical tests were 2-sided, and statistical significance was defined as *p* < 0.05. Findings were reported according to contemporary biomedical reporting conventions, emphasizing effect estimates with 95% confidence intervals rather than relying on *p* values alone. The study reporting was aligned with the Strengthening the Reporting of Observational Studies in Epidemiology guidance for cross-sectional observational research (28).

## 3 Results

### 3.1 Participant characteristics and maintenance discontinuity

**Figure 1:**
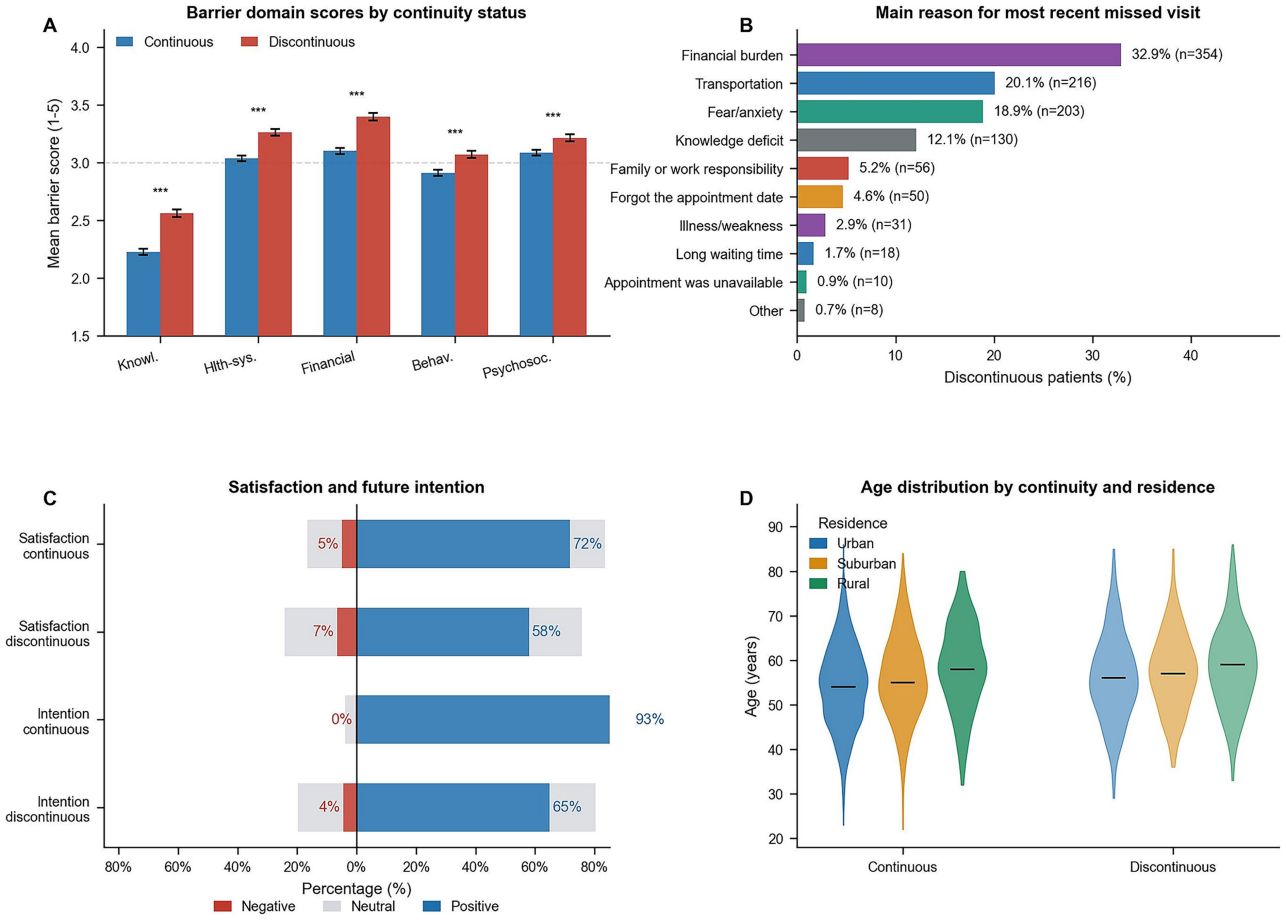
Barrier burden, reasons for discontinuity, and patient-reported experience. **(A)** Barrier-domain scores by maintenance continuity status; higher scores indicate greater barrier burden; error bars represent standard error of the mean; asterisks denote *p* < 0.001 for between-group comparisons. **(B)** Main reason for the most recent missed or delayed maintenance visit among discontinuous patients (*n* = 1,076). **(C)** Satisfaction with outpatient port maintenance services and future on-time maintenance intention by continuity status; response proportions are shown in positive (blue), neutral (light gray), and negative (red) categories. **(D)** Age distribution by maintenance continuity status and place of residence; violin plots represent kernel density estimates of the age distribution within each subgroup; horizontal lines indicate median age. TIVAP, totally implantable venous access port.

A total of 2,710 patients were included in the analytical cohort; the mean age was 56.0 years (SD, 9.5), and 1,683 participants (62.1%) were women. Overall, 1,076 patients (39.7%) reported discontinuous outpatient port maintenance, defined as at least one missed or delayed scheduled maintenance visit during the preceding 6 months, whereas 1,634 patients (60.3%) reported continuous maintenance. Patients with discontinuous maintenance were older than those with continuous maintenance (57.2 vs. 55.2 years; *p* < 0.001) and were more frequently rural residents (29.1% vs. 12.3%), had primary education or below (19.6% vs. 7.0%), and had a monthly household income of <3,000 RMB (21.8% vs. 8.9%; all *p* < 0.001; Table 1; Figure 1D). Insurance type also differed between groups (*p* < 0.001). In contrast, sex, cancer stage, port type, port duration, and prior port-related complication history were not significantly different between continuity groups.

**Table 1:** Key participant characteristics by port maintenance continuity status.

Characteristic	Overall	Continuous	Discontinuous	*p* value
Age, mean (SD), y	56.0 (9.5)	55.2 (9.4)	57.2 (9.4)	<0.001
Sex				
Female	1,683 (62.1)	1,021 (62.5)	662 (61.5)	0.643
Male	1,027 (37.9)	613 (37.5)	414 (38.5)	
Residence				
Urban	1,491 (55.0)	1,047 (64.1)	444 (41.3)	<0.001
Suburban	705 (26.0)	386 (23.6)	319 (29.6)	
Rural	514 (19.0)	201 (12.3)	313 (29.1)	
Education				
Bachelor’s degree or above	813 (30.0)	618 (37.8)	195 (18.1)	<0.001
College/vocational	813 (30.0)	530 (32.4)	283 (26.3)	
Junior/senior high	758 (28.0)	371 (22.7)	387 (36.0)	
Primary or below	326 (12.0)	115 (7.0)	211 (19.6)	
Monthly household income				
>=10,000 RMB	650 (24.0)	496 (30.4)	154 (14.3)	<0.001
6,000–9,999 RMB	867 (32.0)	574 (35.1)	293 (27.2)	
3,000–5,999 RMB	813 (30.0)	419 (25.6)	394 (36.6)	
<3,000 RMB	380 (14.0)	145 (8.9)	235 (21.8)	
Insurance				
UEBMI	1,543 (56.9)	994 (60.8)	549 (51.0)	<0.001
URRBMI	823 (30.4)	435 (26.6)	388 (36.1)	
Commercial/supplementary	203 (7.5)	122 (7.5)	81 (7.5)	
Self-pay/other	141 (5.2)	83 (5.1)	58 (5.4)	
Cancer stage				
I	357 (13.2)	223 (13.6)	134 (12.5)	0.918
II	777 (28.7)	462 (28.3)	315 (29.3)	
III	959 (35.4)	578 (35.4)	381 (35.4)	
IV	503 (18.6)	302 (18.5)	201 (18.7)	
Unknown	114 (4.2)	69 (4.2)	45 (4.2)	
Port type				
Chest wall TIVAP	2,081 (76.8)	1,246 (76.3)	835 (77.6)	0.284
Upper-arm TIVAP	488 (18.0)	308 (18.8)	180 (16.7)	
PICC	141 (5.2)	80 (4.9)	61 (5.7)	
Port duration				
<3 months	366 (13.5)	237 (14.5)	129 (12.0)	0.120
3–6 months	580 (21.4)	356 (21.8)	224 (20.8)	
7–12 months	716 (26.4)	411 (25.2)	305 (28.3)	
>12 months	1,048 (38.7)	630 (38.6)	418 (38.8)	
Prior port complication				
No	2,283 (84.2)	1,379 (84.4)	904 (84.0)	0.833
Yes	427 (15.8)	255 (15.6)	172 (16.0)	

Data are No. (%) unless otherwise indicated. Continuous maintenance was defined as no missed or delayed port maintenance visits in the preceding 6 months; discontinuous maintenance was defined as 1 or more missed or delayed visits. *p* values were calculated using Welch t tests for age and Pearson chi-square tests for categorical variables. UEBMI, Urban Employee Basic Medical Insurance (mandatory payroll-linked scheme for employed urban workers and retirees); URRBMI, Urban–Rural Resident Basic Medical Insurance (voluntary government-subsidized scheme for non-employed urban residents, rural residents, and students); TIVAP, totally implantable venous access port; PICC, peripherally inserted central catheter; SD, standard deviation.

### 3.2 Barrier-domain distribution and scale properties

Financial barriers had the highest overall mean score (3.2 [SD, 0.6]), followed by health-system and psychosocial barriers (both 3.1 [SD, 0.5]), behavioral barriers (3.0 [SD, 0.5]), and knowledge deficit (2.4 [SD, 0.6]). Cronbach alpha ranged from 0.453 to 0.651, reflecting the heterogeneous structure of the barrier constructs, whereas McDonald omega showed more acceptable composite reliability across domains (range, 0.696–0.770). Corrected item-total correlations ranged from 0.21 to 0.42. No interdomain Spearman’s correlation exceeded 0.70, and the maximum variance inflation factor was 2.74, supporting the concurrent inclusion of the five domain scores in the multivariate models. Patients with discontinuous maintenance had higher mean scores across all five barrier domains than those with continuous maintenance (all *p* < 0.001; Figure 1A; Table 2).

**Table 2:** Reliability and distribution of barrier domain scores.

Barrier domain	Items, No.	Score construction	Mean (SD)	Median (IQR)	Cronbach alpha	McDonald omega	Corrected item-total r
Knowledge deficit	6	Reverse-coded knowledge items	2.4 (0.6)	2.33 (2.00–2.67)	0.641	0.770	0.32–0.40
Health-system barriers	8	Positive items reverse coded	3.1 (0.5)	3.12 (2.75–3.50)	0.651	0.766	0.28–0.42
Financial barriers	5	Positive items reverse coded	3.2 (0.6)	3.20 (2.80–3.60)	0.558	0.739	0.29–0.36
Behavioral barriers	5	Barrier-direction items	3.0 (0.5)	3.00 (2.60–3.40)	0.453	0.696	0.21–0.26
Psychosocial barriers	5	Barrier-direction items	3.1 (0.5)	3.20 (2.80–3.40)	0.465	0.700	0.22–0.27
Total barrier score	5 domains	Mean of domain scores	3.0 (0.3)	2.96 (2.79–3.14)			

All scores are oriented so higher values indicate greater barrier burden. The total barrier score was calculated as the simple average of the five derived domain scores. Knowledge items Q14–Q19 were reverse coded. Health-system items Q20, Q21, Q22, and Q27 and financial items Q28 and Q31 were reverse coded. Q38 was excluded from the behavioral score because it measures reminder-message preference rather than barrier burden. Cronbach alpha and McDonald omega were calculated from the scored item sets within each domain. Corrected item-total r gives the observed range across items in each domain. No interdomain Spearman correlation exceeded 0.70, so the domain scores were retained together in the adjusted model. SD, standard deviation; IQR, interquartile range.

### 3.3 Missed visits, delay severity, and port-related service use

**Figure 2:**
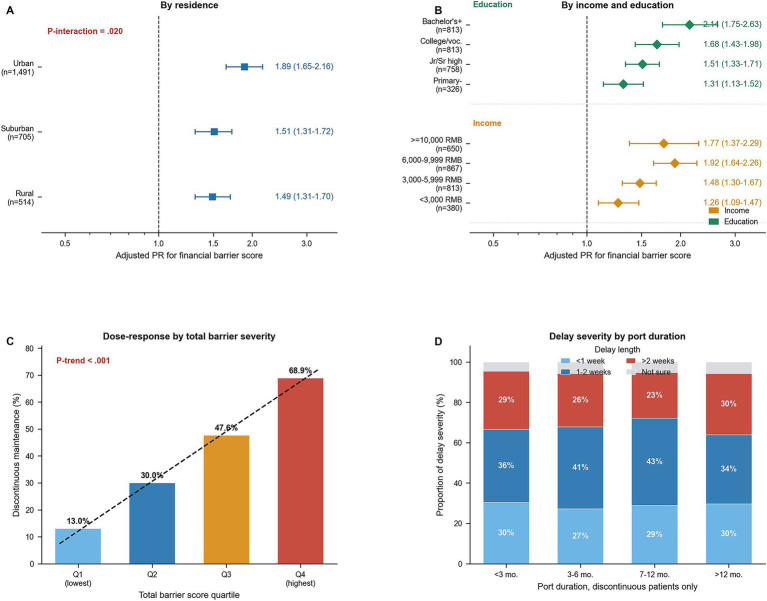
Subgroup variation, dose–response pattern, and delay severity. **(A)** Adjusted prevalence ratios for the financial barrier score by place of residence; estimates are from separate modified Poisson models adjusted for age, sex, and all other barrier-domain scores; *p*-interaction tests the financial barrier score-by-residence interaction. **(B)** Adjusted prevalence ratios for the financial barrier score by income and education subgroups, with the same covariate adjustment; *p*-interaction tests each subgroup interaction independently. **(C)** Prevalence of discontinuous maintenance by total barrier score quartile; the dashed line indicates the linear trend assessed by the Cochran–Armitage test. **(D)** Distribution of delay length among discontinuous patients by port duration stratum; stacked bars show proportions with delays of less than 1 week (light blue), 1–2 weeks (dark blue), more than 2 weeks (red), or uncertain duration (gray). PR, prevalence ratio; CI, confidence interval; RMB, renminbi.

Among the 1,076 patients with discontinuous maintenance, 522 (48.5%) reported one missed or delayed visit, 282 (26.2%) reported two, and 272 (25.3%) reported three or more during the preceding 6 months (Table 3). The longest delay was <1 week in 312 patients (29.0%), 1–2 weeks in 413 (38.4%), and >2 weeks in 292 (27.1%); 59 patients (5.5%) were unsure about the delay duration (Figure 2D). The most common reason for the most recent missed or delayed visit was a financial burden (32.9%; *n* = 354), followed by transportation difficulties (20.1%; n = 216), fear, anxiety, or previous negative healthcare experience (18.9%; *n* = 203), and lack of knowledge about maintenance timing (12.1%; *n* = 130; Figure 1B). Unscheduled port-related hospital visits were more frequent among discontinuous than continuous patients (22.7% vs. 5.5%; *p* < 0.001), as was emergency care or hospitalization for a port-related problem (1.9% vs. 1.0%; *p* = 0.029).

**Table 3:** Maintenance outcomes, delay characteristics, and future intention.

Outcome	Overall	Continuous	Discontinuous	*p* value
Missed/delayed visits in past 6 months				
0	1,634 (60.3)	1,634 (100.0)	0 (0.0)	<0.001
1	522 (19.3)	0 (0.0)	522 (48.5)	
2	282 (10.4)	0 (0.0)	282 (26.2)	
3	272 (10.0)	0 (0.0)	272 (25.3)	
Main reason				
No missed or delayed visit	1,634 (60.3)	1,634 (100.0)	0 (0.0)	<0.001
Financial burden	354 (13.1)	0 (0.0)	354 (32.9)	
Transportation difficulty	216 (8.0)	0 (0.0)	216 (20.1)	
Fear, anxiety, or previous negative experience	203 (7.5)	0 (0.0)	203 (18.9)	
Lack of knowledge about maintenance timing	130 (4.8)	0 (0.0)	130 (12.1)	
Other	173 (6.4)	0 (0.0)	173 (16.1)	
Longest delay				
No delay	1,634 (60.3)	1,634 (100.0)	0 (0.0)	<0.001
<1 week	312 (11.5)	0 (0.0)	312 (29.0)	
1–2 weeks	413 (15.2)	0 (0.0)	413 (38.4)	
>2 weeks	292 (10.8)	0 (0.0)	292 (27.1)	
Not sure	59 (2.2)	0 (0.0)	59 (5.5)	
Unscheduled port-related visit				
No	2,311 (85.3)	1,505 (92.1)	806 (74.9)	<0.001
Yes	334 (12.3)	90 (5.5)	244 (22.7)	
Not sure	65 (2.4)	39 (2.4)	26 (2.4)	
Emergency/hospitalization				
No	2,645 (97.6)	1,595 (97.6)	1,050 (97.6)	0.029
Yes	37 (1.4)	17 (1.0)	20 (1.9)	
Not sure	28 (1.0)	22 (1.3)	6 (0.6)	
Satisfaction				
Very satisfied	514 (19.0)	364 (22.3)	150 (13.9)	<0.001
Satisfied	1,282 (47.3)	808 (49.4)	474 (44.1)	
Neutral	764 (28.2)	382 (23.4)	382 (35.5)	
Dissatisfied	103 (3.8)	54 (3.3)	49 (4.6)	
Very dissatisfied	47 (1.7)	26 (1.6)	21 (2.0)	
Future on-time intention				
Definitely will attend	1,128 (41.6)	910 (55.7)	218 (20.3)	<0.001
Probably will attend	1,085 (40.0)	605 (37.0)	480 (44.6)	
Unsure	443 (16.3)	113 (6.9)	330 (30.7)	
Probably will not attend	54 (2.0)	6 (0.4)	48 (4.5)	

Data are No. (%). Less frequent reasons for missed/delayed visits were combined as other to keep the main table readable. *p* values were calculated using Pearson chi-square tests comparing continuous and discontinuous maintenance groups.

### 3.4 Satisfaction and future maintenance intention

Patient-reported experience differed substantially by continuity status. Satisfied or very satisfied responses were reported by 624 discontinuous patients (58.0%) compared with 1,172 continuous patients (71.7%; *p* < 0.001). Future intention to attend maintenance visits on time showed a larger difference: probable or definite future attendance was reported by 698 discontinuous patients (64.9%) compared with 1,515 continuous patients (92.7%; *p* < 0.001). Uncertainty about future attendance was also more common among discontinuous patients (30.7% vs. 6.9%; Figure 1C).

### 3.5 Predictors of discontinuous maintenance

**Figure 3:**
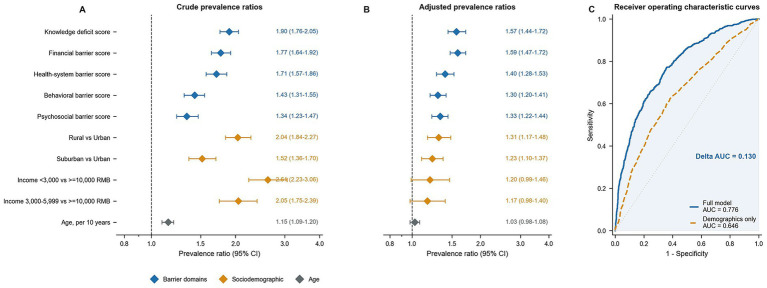
Predictors and discrimination of discontinuous maintenance. **(A)** Crude prevalence ratios from univariable modified Poisson regression for all prespecified predictors of discontinuous port maintenance, including barrier-domain scores, residence, income, and age **(B)** Fully adjusted prevalence ratios from modified Poisson regression with robust standard errors. **(C)** Receiver operating characteristic curves comparing the demographics-only model with the full model including clinical and barrier-domain variables. PR, prevalence ratio; aPR, adjusted prevalence ratio; CI, confidence interval; AUC, area under the receiver operating characteristic curve.

In the univariable modified Poisson models, all five barrier domains were associated with discontinuous maintenance. Knowledge deficit showed the strongest crude association (PR, 1.90; 95% CI, 1.76–2.05), followed by financial barriers (PR, 1.77; 95% CI, 1.64–1.92), health system barriers (PR, 1.71; 95% CI, 1.57–1.86), behavioral barriers (PR, 1.43; 95% CI, 1.31–1.55), and psychosocial barriers (PR, 1.34; 95% CI, 1.23–1.47; all *p* < 0.001; Figure 3A; Table 4). The total barrier score showed a strong cumulative association with discontinuity (PR, 8.27; 95% CI, 7.08–9.67). Sociodemographic gradients were also evident before adjustment, particularly for rural residence (PR, 2.04; 95% CI, 1.84–2.27), monthly income <3,000 RMB (PR, 2.61; 95% CI, 2.23–3.06), and primary education or below (PR, 2.70; 95% CI, 2.33–3.12).

**Table 4:** Univariable associations with discontinuous port maintenance.

Predictor	Crude PR (95% CI)	*p* value
Age, per 10 years	1.15 (1.09–1.20)	<0.001
Knowledge deficit score	1.90 (1.76–2.05)	<0.001
Health-system barrier score	1.71 (1.57–1.86)	<0.001
Financial barrier score	1.77 (1.64–1.92)	<0.001
Behavioral barrier score	1.43 (1.31–1.55)	<0.001
Psychosocial barrier score	1.34 (1.23–1.47)	<0.001
Total barrier score	8.27 (7.08–9.67)	<0.001
Residence: Suburban vs. Urban	1.52 (1.36–1.70)	<0.001
Residence: Rural vs. Urban	2.04 (1.84–2.27)	<0.001
Education: College/vocational vs. Bachelor’s degree or above	1.45 (1.24–1.69)	<0.001
Education: Junior/senior high vs. Bachelor’s degree or above	2.13 (1.85–2.45)	<0.001
Education: Primary or below vs. Bachelor’s degree or above	2.70 (2.33–3.12)	<0.001
Income: 6,000–9,999 RMB vs. >=10,000 RMB	1.43 (1.21–1.68)	<0.001
Income: 3,000–5,999 RMB vs. >=10,000 RMB	2.05 (1.75–2.39)	<0.001
Income: <3,000 RMB vs. >=10,000 RMB	2.61 (2.23–3.06)	<0.001

PR indicates prevalence ratio. Each row is a separate modified Poisson regression model with robust standard errors. Barrier scores are modeled per 1-point increase on the 1–5 scale. The table is restricted to major predictors to reduce width and redundancy.

After adjusting for sociodemographic, clinical, device-related, and barrier domain variables, all five barrier domains remained independently associated with discontinuous maintenance. Financial barriers were the strongest adjusted predictors (aPR, 1.59; 95% CI, 1.47–1.72), followed by knowledge deficits (aPR, 1.57; 95% CI, 1.44–1.72), health system barriers (aPR, 1.40; 95% CI, 1.28–1.53), psychosocial barriers (aPR, 1.33; 95% CI, 1.22–1.44), and behavioral barriers (aPR, 1.30; 95% CI, 1.20–1.41; all *p* < 0.001; Figure 3B, Table 5). Suburban and rural residences also remained independently associated with discontinuity (aPR, 1.23; 95% CI, 1.10–1.37; and aPR, 1.31; 95% CI, 1.17–1.48, respectively), whereas age, education, and income were attenuated after barrier adjustment. Model discrimination improved from an AUC of 0.646 in the demographics-only model to 0.776 in the full model (ΔAUC = 0.130; Figure 3C).

**Table 5:** Adjusted associations with discontinuous port maintenance.

Predictor	Adjusted PR (95% CI)	*p* value
Age, per 10 years	1.03 (0.98–1.08)	0.297
Suburban vs. reference	1.23 (1.10–1.37)	<0.001
Rural vs. reference	1.31 (1.17–1.48)	<0.001
Junior/senior high vs. reference	1.17 (0.99–1.38)	0.059
Primary or below vs. reference	1.08 (0.88–1.32)	0.462
3,000–5,999 RMB vs. reference	1.17 (0.98–1.40)	0.075
<3,000 RMB vs. reference	1.20 (0.99–1.46)	0.066
Knowledge deficit score	1.57 (1.44–1.72)	<0.001
Health-system barrier score	1.40 (1.28–1.53)	<0.001
Financial barrier score	1.59 (1.47–1.72)	<0.001
Behavioral barrier score	1.30 (1.20–1.41)	<0.001
Psychosocial barrier score	1.33 (1.22–1.44)	<0.001

Adjusted PR indicates adjusted prevalence ratio from one modified Poisson model with robust standard errors. The table displays the main predictors; the model additionally adjusted for sex, insurance type, prior port complication, primary cancer diagnosis, treatment status, cancer stage, port type, and port duration. Reference groups were urban residence, bachelor’s degree or above, monthly household income >=10,000 RMB, female sex, UEBMI insurance, no prior port complication, breast cancer, chemotherapy, stage I, chest wall TIVAP, and port duration <3 months. Maximum variance inflation factor was 2.74. aPR, adjusted prevalence ratio; CI, confidence interval; PR, proportional odds ratio; RMB, renminbi.

### 3.6 Subgroup, dose–response, and severity analyses

The association between financial barriers and discontinuous maintenance varied by residence, income, and education. Financial barriers were associated with discontinuity in urban (aPR, 1.89; 95% CI, 1.65–2.16), suburban (aPR, 1.51; 95% CI, 1.31–1.72), and rural (aPR, 1.49; 95% CI, 1.31–1.70; *p* for interaction = 0.020) patients (Figure 2A; Table 6). A significant interaction was also observed by income and education (both *p* for interaction = 0.002; Figure 2B), although all subgroup-specific associations remained statistically significant. A graded dose–response pattern was observed across quartiles of the total barrier score, with discontinuity increasing from 13.0% in Q1 to 30.0% in Q2, 47.6% in Q3, and 68.9% in Q4 (*p* for trend < 0.001; Figure 2C). This more-than-fivefold increase from the lowest to the highest quartile demonstrates that the simultaneous accumulation of multiple barrier types—rather than any single domain in isolation—is strongly associated with the greatest disruption in port maintenance continuity, supporting a cumulative burden model of access failure. In the ordinal model for missed-visit severity, the strongest predictors were knowledge deficit (POR, 2.50; 95% CI, 2.15–2.90), financial barriers (POR, 2.30; 95% CI, 1.99–2.66), and health system barriers (POR, 2.20; 95% CI, 1.87–2.59), followed by psychosocial and behavioral barriers (Table 6).

**Table 6:** Subgroup and severity analyses.

Variable	Level	No.	Discontinuous, %	Estimate (95% CI)	*p* value	Interaction *p*
A. Subgroup analyses: Association between financial barrier score and discontinuous port maintenance						
Residence	Urban	1,491	29.8	1.89 (1.65–2.16)	<0.001	0.020
	Suburban	705	45.2	1.51 (1.31–1.72)	<0.001	
	Rural	514	60.9	1.49 (1.31–1.70)	<0.001	
Income	>=10,000 RMB	650	23.7	1.77 (1.37–2.29)	<0.001	0.002
	6,000–9,999 RMB	867	33.8	1.92 (1.64–2.26)	<0.001	
	3,000–5,999 RMB	813	48.5	1.48 (1.30–1.67)	<0.001	
	<3,000 RMB	380	61.8	1.26 (1.09–1.47)	0.002	
Education	Bachelor’s degree or above	813	24.0	2.14 (1.75–2.63)	<0.001	0.002
	College/vocational	813	34.8	1.68 (1.43–1.98)	<0.001	
	Junior/senior high	758	51.1	1.51 (1.33–1.71)	<0.001	
	Primary or below	326	64.7	1.31 (1.13–1.52)	<0.001	
B. Ordinal severity analyses: Predictors of missed-visit frequency (0, 1, 2, or ≥3 missed visits in the preceding 6 months)						
Age, per 10 years				1.09 (1.00–1.19)	0.040	
Knowledge deficit				2.50 (2.15–2.90)	<0.001	
Health-system				2.20 (1.87–2.59)	<0.001	
Financial				2.30 (1.99–2.66)	<0.001	
Behavioral				1.62 (1.39–1.89)	<0.001	
Psychosocial				1.63 (1.40–1.90)	<0.001	

Subgroup rows show adjusted PRs for discontinuous maintenance per 1-point increase in financial barrier score, adjusted for age and sex. Interaction *p* values test financial barrier score by subgroup interaction. Ordinal severity rows show proportional odds ratios for greater missed or delayed visit frequency during the preceding 6 months (0, 1, 2, or ≥3 visits), modeled as an ordinal outcome and adjusted for age and all five barrier-domain scores. POR, proportional odds ratio; CI, confidence interval.

### 3.7 Barrier domains associated with satisfaction and future intention

**Figure 4:**
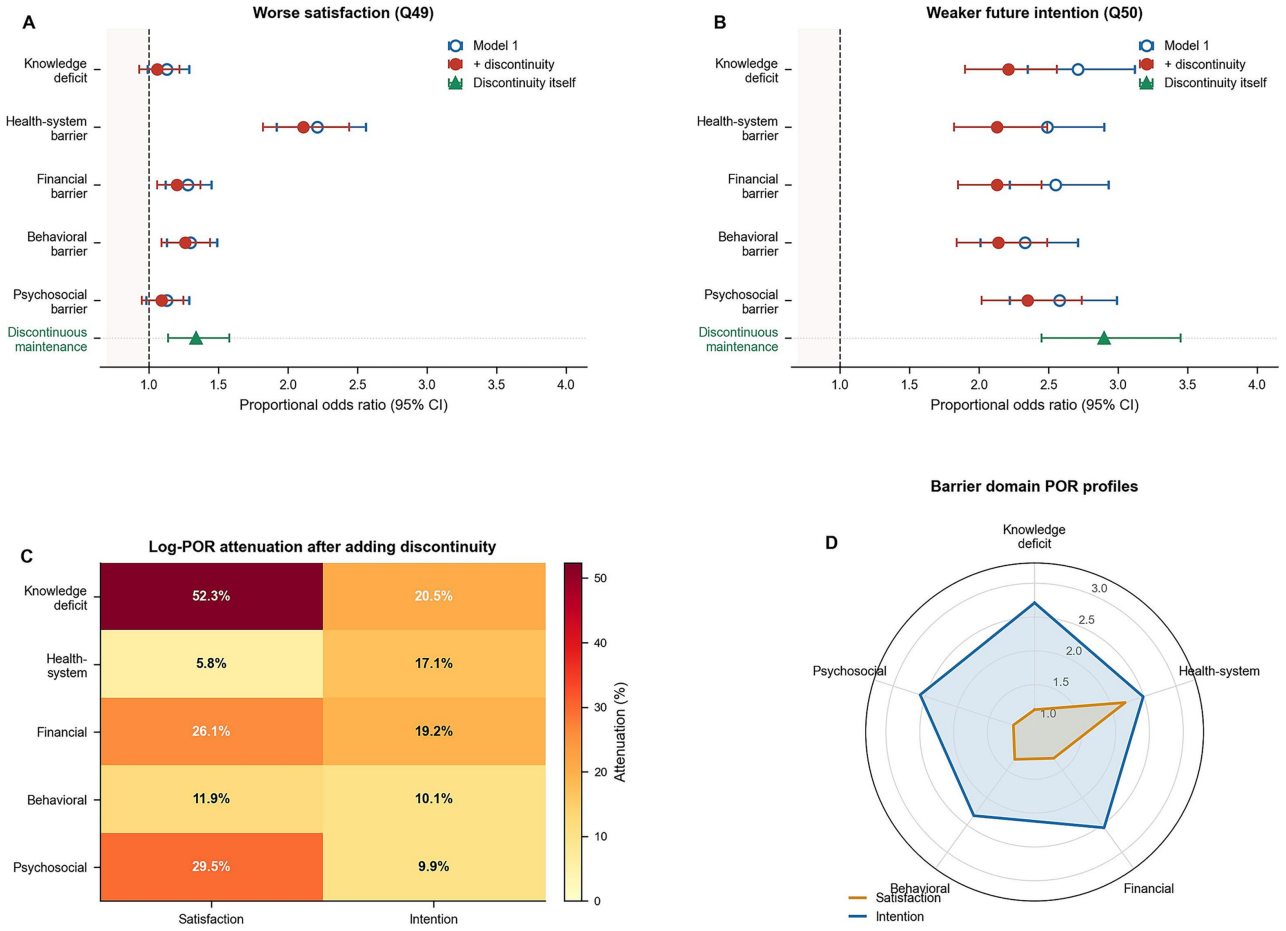
Barrier-domain associations with satisfaction and future intention. **(A)** Ordinal-model estimates for worse satisfaction. **(B)** Ordinal-model estimates for weaker future on-time maintenance intention. **(C)** Percentage attenuation of the log-proportional odds ratio for each barrier domain after adding discontinuous maintenance to the satisfaction and future-intention ordinal models; darker shading indicates greater attenuation, suggesting that the barrier’s effect on the outcome is more closely mediated through maintenance discontinuity. **(D)** Radar chart comparing barrier-domain proportional odds ratio profiles for worse satisfaction (orange) and weaker future on-time maintenance intention (blue), illustrating the divergent domain weighting between these two patient-reported outcomes. POR, proportional odds ratio; CI, confidence interval.

Ordinal models showed distinct profiles for satisfaction and future intention (Table 7; Figures 4A–D). Lower satisfaction was dominated by health system barriers (POR, 2.21; 95% CI, 1.92–2.56), with smaller associations for behavioral barriers (POR, 1.30; 95% CI, 1.13–1.49) and financial barriers (POR, 1.28; 95% CI, 1.12–1.45), as shown in Figure 4A. Knowledge deficits and psychosocial barriers were not significant predictors of lower satisfaction. After adding discontinuous maintenance, health system barriers remained strongly associated with lower satisfaction (POR, 2.11; 95% CI, 1.82–2.44), and discontinuity itself was independently associated with lower satisfaction (POR, 1.34; 95% CI, 1.14–1.58; *p* < 0.001). Attenuation was largest for knowledge deficits (52.3%) and minimal for health system barriers (5.8%), an exploratory pattern consistent with the possibility that knowledge-related dissatisfaction may be more closely associated with maintenance discontinuity, whereas system-related dissatisfaction appeared more directly associated with the service environment regardless of whether visits were missed; these patterns do not establish mediated causal pathways.

**Table 7:** Ordinal models for worse satisfaction and weaker future intention.

Outcome	Model	Predictor	POR (95% CI)	*p* value
Satisfaction	Model 1	Knowledge deficit	1.13 (0.99–1.29)	0.070
Satisfaction	Model 1	Health-system barrier	2.21 (1.92–2.56)	<0.001
Satisfaction	Model 1	Financial barrier	1.28 (1.12–1.45)	<0.001
Satisfaction	Model 1	Behavioral barrier	1.30 (1.13–1.49)	<0.001
Satisfaction	Model 1	Psychosocial barrier	1.13 (0.98–1.29)	0.085
Satisfaction	+ discontinuity	Knowledge deficit	1.06 (0.93–1.22)	0.392
Satisfaction	+ discontinuity	Health-system barrier	2.11 (1.82–2.44)	<0.001
Satisfaction	+ discontinuity	Financial barrier	1.20 (1.06–1.37)	0.006
Satisfaction	+ discontinuity	Behavioral barrier	1.26 (1.09–1.44)	0.001
Satisfaction	+ discontinuity	Psychosocial barrier	1.09 (0.95–1.25)	0.206
Satisfaction	+ discontinuity	Discontinuous maintenance	1.34 (1.14–1.58)	<0.001
Future intention	Model 1	Knowledge deficit	2.71 (2.35–3.12)	<0.001
Future intention	Model 1	Health-system barrier	2.49 (2.13–2.90)	<0.001
Future intention	Model 1	Financial barrier	2.55 (2.22–2.93)	<0.001
Future intention	Model 1	Behavioral barrier	2.33 (2.01–2.71)	<0.001
Future intention	Model 1	Psychosocial barrier	2.58 (2.22–2.99)	<0.001
Future intention	+ discontinuity	Knowledge deficit	2.21 (1.90–2.56)	<0.001
Future intention	+ discontinuity	Health-system barrier	2.13 (1.82–2.49)	<0.001
Future intention	+ discontinuity	Financial barrier	2.13 (1.85–2.45)	<0.001
Future intention	+ discontinuity	Behavioral barrier	2.14 (1.84–2.49)	<0.001
Future intention	+ discontinuity	Psychosocial barrier	2.35 (2.02–2.74)	<0.001
Future intention	+ discontinuity	Discontinuous maintenance	2.90 (2.45–3.45)	<0.001

POR indicates proportional odds ratio. Outcomes were coded so higher categories represent worse satisfaction or weaker future on-time maintenance intention. Model 1 included age and the five barrier domain scores. The discontinuity model added derived discontinuous maintenance. These are exploratory attenuation models, not causal mediation analyses.

Weaker future maintenance intention was strongly associated with all five domains: knowledge deficit (POR, 2.71; 95% CI, 2.35–3.12), psychosocial barriers (POR, 2.58; 95% CI, 2.22–2.99), financial barriers (POR, 2.55; 95% CI, 2.22–2.93), health system barriers (POR, 2.49; 95% CI, 2.13–2.90), and behavioral barriers (POR, 2.33; 95% CI, 2.01–2.71; all *p* < 0.001). After adding discontinuous maintenance, all domains remained significant, and discontinuity itself was the strongest single predictor of weaker future intention (POR, 2.90; 95% CI, 2.45–3.45; *p* < 0.001). Overall, discontinuous port maintenance was common and was independently associated with financial, knowledge-related, health system, behavioral, and psychosocial barriers, identifying several modifiable targets for outpatient oncology service improvement.

## 4 Discussion

This hospital-based observational study identified a substantial continuity gap in outpatient port maintenance among cancer patients. Nearly two in five participants reported at least one missed or delayed maintenance visit within the preceding 6 months, and discontinuity was not explained by cancer stage, port type, port duration, or previous port-related complications. Instead, discontinuous maintenance clustered around modifiable access and self-management barriers, particularly the financial burden, knowledge deficit, health system constraints, transportation difficulties, and psychosocial distress. These findings extend the vascular-access literature beyond device performance and complication surveillance by showing that the practical organization of maintenance care is a central determinant of whether patients can preserve safe long-term port use.

The present results are consistent with contemporary vascular-access guidance, which conceptualizes totally implantable venous access ports as long-term supportive-care devices requiring standardized maintenance, patient education, complication recognition, and coordinated follow-up rather than simple technical flushing procedures (4, 7, 29). The observed prevalence of discontinuity is clinically important because more than one-quarter of discontinuous patients reported delays exceeding 2 weeks, and one-quarter reported three or more missed or delayed visits. Although delayed maintenance is not equivalent to catheter malfunction, the higher frequency of unscheduled port-related hospital visits among discontinuous patients suggests that maintenance disruption may be a measurable marker of avoidable service use, symptom escalation, or perceived device insecurity.

The association between discontinuous maintenance and unscheduled port-related care aligns with studies showing that central venous access devices remain vulnerable to occlusion, infection, thrombosis, mechanical dysfunction, and complication-related removal throughout their life cycle (6, 30–32). However, these results must also be interpreted in light of recent evidence suggesting that structured extension of flushing intervals may be safe in selected patients, particularly after completion or interruption of active therapy (11, 12, 33–35). At first glance, this literature may appear to contradict the present finding that missed or delayed visits were associated with poorer patient-reported outcomes and greater unscheduled care. This apparent discrepancy may be accounted for by a critical distinction: protocolized interval extension is a planned, risk-stratified care model, whereas discontinuous maintenance in this study reflected unplanned non-attendance associated with financial, transportation, knowledge, behavioral, and psychosocial barriers. Therefore, these findings do not argue against evidence-based interval extension; rather, they indicate that any extended-maintenance strategy must be accompanied by clear patient education, triage pathways, reminder systems, and mechanisms for rapid assessment when warning symptoms occur.

The data also support the growing emphasis on patient self-management in TIVAP care. A recent Chinese scale-development study for TIVAP self-management behavior reflects the increasing recognition that patients require structured knowledge, symptom awareness, and maintenance-related behavioral skills after discharge (20). In the present study, knowledge deficit was one of the strongest adjusted predictors of discontinuity and the strongest predictor of missed-visit severity. This pattern is biologically and behaviorally plausible because inadequate knowledge may reduce perceived urgency after chemotherapy is paused or completed, obscure warning signs, and impair patients’ ability to identify where and when maintenance should be performed. In contrast to purely procedural models of port care, these findings suggest that maintenance continuity is partly a health-literacy-sensitive behavior embedded within the broader outpatient oncology pathway.

The financial burden was the most frequently reported reason for the most recent missed or delayed visit and the strongest adjusted predictor of discontinuous maintenance. This finding is consistent with the expanding oncology financial-toxicity literature, which shows that cancer-related costs can affect adherence, quality of life, psychological wellbeing, and care-seeking behavior even in health systems with broad insurance coverage (36–43). Similarly, studies shown that financial toxicity is common among patients with cancer and is shaped by multilevel factors, including household income, insurance coverage, disease burden, treatment cost, and regional economic context (39, 44–47). The present study contributes a more specific service-continuity endpoint by showing that the financial burden extends beyond treatment affordability and affects routine supportive care maintenance.

The attenuation of income and education after adjustment for barrier-domain scores is also important. Rather than contradicting socioeconomic-disparity research, this pattern suggests that the measured barriers may partially explain how socioeconomic disadvantage translates into missed maintenance. Low income and lower education were strongly associated with discontinuity in crude analyses; however, their independent effects weakened after the inclusion of financial, knowledge, health system, behavioral, and psychosocial domains. This finding is consistent with an associational pattern in which socioeconomic position may operate through concrete and potentially modifiable determinants, including out-of-pocket costs, indirect travel costs, appointment navigation, health literacy, caregiver availability, and the perceived benefit of maintenance (47–50).

Transportation difficulties were the second most common reason for discontinuity, and rural residence remained independently associated with missed or delayed maintenance after full adjustment. This result is consistent with recent oncology access literature, which identifies transportation as an underrecognized but modifiable determinant of delayed appointments, missed care, reduced access to specialized oncology services, and rural cancer disparities (51–54). The persistence of rural and suburban effects after adjustment suggests that geographic access may not be fully captured by individual-level barrier scores. Distance, transport reliability, referral fragmentation, availability of trained maintenance clinics, and the concentration of vascular access expertise in tertiary centers may all contribute to residual residence-based inequity.

A major finding was the distinct profile of predictors for satisfaction versus future maintenance intention. Lower satisfaction was dominated by health-system barriers, whereas weaker future intention was strongly associated with all five domains and was most strongly linked to prior discontinuity. This pattern is consistent with evidence that outpatient satisfaction is shaped by access, waiting time, appointment efficiency, communication, navigation, and perceived service coordination (55–57). In this study, the limited attenuation of health-system barriers after adding discontinuity indicates that dissatisfaction was not merely a consequence of missed visits; rather, patients appeared to evaluate the service environment directly. Long waiting times, difficulty obtaining appointments, limited-service hours, poor departmental coordination, and uncertainty about where to seek maintenance may therefore undermine satisfaction even among patients who eventually attend.

The substantially higher proportion of uncertain future attenders among discontinuous compared with continuous patients (30.7% vs. 6.9%) is consistent with the possibility that prior maintenance disruption may be associated with reduced confidence in sustaining future attendance. In contrast, weaker future intention appears to reflect cumulative vulnerability rather than a single domain. Knowledge deficit, financial barriers, health-system barriers, psychosocial barriers, and behavioral barriers all showed strong associations with weaker intention, and discontinuity itself was the strongest single predictor after adjustment. This association is consistent with a reinforcing cycle in which patients who miss maintenance may become less confident, less satisfied, or less motivated to maintain future attendance, especially when missed visits are accompanied by fear, uncertainty, cost concerns, or negative prior experiences. Because the cross-sectional design cannot establish whether psychosocial responses preceded or followed maintenance disruption, longitudinal follow-up across successive maintenance cycles would be required to confirm this hypothesized temporal pattern. From a public-health perspective, this cycle is important because future intention may represent an early warning indicator of subsequent disengagement from supportive oncology care (57–59).

The graded increase in discontinuity across total barrier-score quartiles provides strong internal coherence for the study findings. Discontinuity increased from 13.0% in the lowest quartile to 68.9% in the highest quartile, supporting a cumulative barrier-burden model rather than a single-cause explanation. The subgroup findings further suggest that financial barriers operate differently across social groups. The adjusted association between financial barriers and discontinuity was strongest among urban patients and patients with higher education, although the absolute discontinuity burden was higher among rural, lower-income, and lower-education groups. This pattern should not be interpreted as financial barriers being unimportant for disadvantaged groups. A more plausible explanation is that disadvantaged groups may already have high baseline discontinuity due to multiple overlapping structural barriers, producing a relative-effect ceiling, whereas among relatively advantaged groups financial strain may more clearly distinguish those who become discontinuous from those who remain adherent. These findings are also compatible with financial-toxicity frameworks that distinguish absolute burden, subjective distress, coping capacity, and downstream behavioral consequences (36, 37, 40, 60). Patients with higher levels of education or urban residence may have greater awareness of maintenance requirements but may still delay care when indirect costs, work disruption, travel time, or repeated outpatient charges accumulate. Conversely, rural and lower-income patients may experience simultaneous transportation, insurance, knowledge, and service-availability barriers, so the relative effect of any single barrier domain may appear smaller despite greater absolute vulnerability.

The present findings should be interpreted within a continuity-of-care framework rather than a narrow adherence framework. Although missed port maintenance may appear to be an individual behavioral outcome, the pattern observed in this study indicates that discontinuity emerges from the interaction between patient knowledge, household resources, transport feasibility, appointment systems, psychosocial readiness, and the organization of outpatient oncology services. This interpretation is important because it shifts the prevention strategy from patient reminder alone to a multicomponent access intervention. Conversely, a well-organized service may be insufficient if patients believe that maintenance is unnecessary once chemotherapy is paused or completed. Therefore, a sustainable intervention should address both demand-side and supply-side determinants (61, 62).

The findings also suggest that port maintenance should be embedded within survivorship and chronic cancer-care models. Increasing cancer survival has expanded the number of patients living with long-term venous access devices, and the safety of these devices depends on repeated low-intensity encounters rather than episodic high-intensity treatment alone. This care pattern resembles chronic disease management, in which continuity, recall systems, patient education, and community coordination determine outcomes. Accordingly, oncology services should regard port-maintenance non-attendance as a sentinel event that indicates breakdown in the supportive-care pathway. Routine dashboards could monitor missed appointments, delay duration, repeated non-attendance, and unscheduled port-related visits, allowing early intervention at the patient and service levels (63–65).

The distinction between satisfaction and future intention further enhances the policy relevance of the findings. Satisfaction was primarily shaped by health-system barriers, whereas future intention reflected a broader accumulation of barriers and prior discontinuity. This divergence suggests that improving clinic efficiency and coordination may directly improve patient experience, but sustaining future attendance will require a more comprehensive strategy that also addresses knowledge, fear, affordability, and behavioral support (66, 67). In practical terms, service redesign should not rely solely on increasing appointment availability; it should combine education, navigation, reminder systems, financial counseling, and psychosocial reassurance. Such integration is especially important in health systems where tertiary oncology centers remain the primary source of vascular-access expertise, but patients increasingly require long-term outpatient care closer to home. Equity should be central to this redesign. The persistence of rural and suburban effects after adjustment indicates that geographic disadvantage may remain, even when individual barriers are considered. This residual effect probably reflects unmeasured contextual factors, including the local availability of trained nurses, referral networks, regional reimbursement practices, and transportation infrastructure. Therefore, community-based maintenance expansion should not be implemented merely as decentralization; it requires standardized competency training, quality control, documentation systems, escalation protocols, and linkages with tertiary oncology teams. Without these safeguards, decentralization may reduce the travel burden while introducing variation in maintenance quality (62, 68). A coordinated hub-and-spoke model may therefore offer a balanced approach, with tertiary centers providing protocols, training, audits, and complication consultations, and community clinics delivering routine maintenance for clinically stable patients.

The findings support a shift from procedure-centered port maintenance to continuity-centered supportive oncology care. At the clinical level, every patient discharged with a port should receive standardized education on maintenance timing, warning symptoms, the consequences of delay, and the correct department or contact pathway for urgent concerns. Education should be reinforced when chemotherapy is paused or completed because patients may incorrectly infer that port maintenance is no longer necessary. Written instructions, mobile reminders, nurse-led telephone follow-up, and brief teach-back methods may be particularly useful for patients with lower levels of education or previous missed visits. The findings also suggest that health systems should monitor missed port maintenance visits as a quality indicator. A missed maintenance visit is not only a marker of maintenance discontinuity but also a signal of potential access failure. Patients with repeated delays, high barrier scores, or weak future intentions could be prioritized for nurse navigation, reminder escalation, financial counseling, or telephonic assessment. Such a risk-stratified approach would align vascular access maintenance with broader public health goals of continuity, equity, and avoidable hospital use reduction.

This study has several strengths, including a large analytical sample, a clinically specific continuity outcome, domain-based measurement of modifiable barriers, use of prevalence ratios for a common outcome, evaluation of scale reliability, assessment of multicollinearity, subgroup analyses, and linkage of maintenance discontinuity with patient-reported satisfaction, future intention, and unscheduled service use. The study also addresses an underexamined public health dimension of oncology vascular-access care by focusing on outpatient continuity rather than only insertion success or device complications. This study has several limitations. First, the cross-sectional design precludes causal inference and cannot establish temporal ordering among barriers, missed visits, satisfaction, and future intentions; this constraint is particularly salient for the psychosocial domain, where anxiety, fear, and negative prior healthcare experiences were modeled as antecedent barriers but may partly develop as consequences of missed visits or adverse unscheduled care encounters, and longitudinal cohort studies are required to clarify directionality. Second, maintenance behavior was self-reported over a 6-month recall window without structured bias-reduction procedures such as anonymous response formats or cross-validation with appointment records, and patients completing the questionnaire in a clinical setting may have underreported missed visits to avoid perceived clinical judgment, which would tend to underestimate the true discontinuity prevalence; electronic health record linkage to benchmark recall accuracy was not performed and should be incorporated in future studies. Third, although the questionnaire was administered by trained study personnel who provided verbal clarification when needed, patients with severe literacy limitations may have been underrepresented, a standardized readability assessment was not applied, median pilot-test completion time was not formally recorded, and the barrier-domain items did not include open-ended write-in options, which may have prevented capture of unanticipated barriers outside the prespecified domain structure. Fourth, the study was conducted at a single tertiary referral center where patients are likely disproportionately urban, better insured, and living in closer proximity to care than the broader Chinese cancer population, so the observed discontinuity prevalence and barrier burden may underestimate the true population-level figures, and multisite studies incorporating primary care, county hospital, and rural oncology settings are needed to establish representative estimates. Fifth, Cronbach alpha values were modest for several barrier domains (range, 0.453–0.651), reflecting the intentionally heterogeneous construct structure; reliability was evaluated jointly using McDonald’s omega, corrected item-total correlations, and conceptual coherence, and domain scores should be interpreted as approximate composite measures rather than psychometrically mature scales. Sixth, systematic screening logs were not maintained prospectively, precluding construction of a complete STROBE-compliant participant flow diagram, and unscheduled port-related visits and emergency care were patient-reported outcomes rather than adjudicated clinical events; future studies should prospectively record all screening contacts and link questionnaire data with clinical records, port-function assessments, and complication diagnoses. Seventh, 141 participants (5.2%) had peripherally inserted central catheters rather than totally implantable ports; because PICCs typically require more frequent maintenance visits and may involve closer clinical monitoring than chest-wall TIVAPs, the direction of any resulting bias is uncertain, though the small proportion makes a material effect on the main estimates unlikely, and future studies should enroll and analyze implantable-port and PICC cohorts separately.

## 5 Conclusion

Discontinuous outpatient infusion-port maintenance was frequent among patients with cancer and reflected a multidimensional continuity-of-care problem rather than a simple adherence failure. By identifying financial burden, knowledge deficit, health-system barriers, psychosocial concerns, and behavioral constraints as independent determinants of missed or delayed maintenance, this study addresses an important gap in supportive oncology research, where port care has traditionally been evaluated through device complications or flushing protocols rather than access continuity. The strong dose–response relationship between cumulative barrier burden and discontinuity, together with higher unscheduled port-related service use and weaker future attendance intention, suggests that missed maintenance visits may serve as an early warning indicator of supportive-care disengagement. These findings support a shift toward integrated, equity-oriented port-maintenance pathways that combine structured education, appointment coordination, reminder systems, financial navigation, transportation support, and hospital–community linkage to improve long-term vascular-access safety and continuity of cancer care.

## Data Availability

The raw data supporting the conclusions of this article will be made available by the authors, without undue reservation.
